# Changes in physicochemical properties and bacterial communities in aged Korean native cattle beef during cold storage

**DOI:** 10.1002/fsn3.2864

**Published:** 2022-04-01

**Authors:** Sun Hye Hwang, JaeHwan Lee, Tae Gyu Nam, Minseon Koo, Yong Sun Cho

**Affiliations:** ^1^ 71645 Food Analysis Center Korea Food Research Institute Wanju‐gun Korea; ^2^ Department of Food Science and Biotechnology Sungkyunkwan University Suwon Korea; ^3^ 34979 Major of Food Science and Biotechnology Division of Bio‐Convergence Kyonggi University Suwon Korea

**Keywords:** aged beef, free amino acid, microorganism, volatile basic nitrogen, volatile compound

## Abstract

During refrigerated storage, aged beef is liable to undergo alterations in its physicochemical properties. This study aimed to evaluate changes in the community of microorganisms, volatile compounds, and amino acids in aged beef under cold storage conditions. In addition, volatile basic nitrogen (VBN) values were measured to determine the putrefaction degree. Raw‐, dry‐, and wet‐aged beef were stored at 4°C for 21 days. The initial pH of beef under the three conditions ranged from 5.52 to 5.60 and decreased from 5.04 to 5.33 over time. After 21 days, VBN values ranged 20.53–22.59 mg/100 g, which exceeded the standard of spoilage (20 mg/100 g) in the Korean Food Code. As time passed, numbers of psychrophilic and lactic acid bacteria increased in the raw beef. In contrast, number of mesophilic, psychrophilic, and lactic acid bacteria decreased in dry‐ and wet‐aged beef. Among the volatile substances and amino acids, 2,3‐butanedione, 2‐butanone, tyrosine, and arginine contributed the most to the high VBN levels in aged beef, where the VBN was 21 mg/100 g at 21 days, which was beyond the acceptable limit. In conclusion, clear alterations were observed in the physicochemical properties and microorganism communities in cold‐stored aged beef, providing basic information that could benefit the beef industry and boost consumer acceptance.

## Graphical abstract

Microbial, physicochemical changes due to refrigeration storage of aged Korean native cattle.

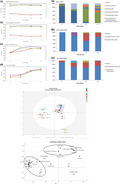

## 1 INTRODUCTION

As livestock grows old, the muscle becomes less fatty as fat deposits shrink, and the connective tissue, such as the fascia connecting the muscles, and the muscle fibers do not dissolve as readily (Prache et al., 2020; Utama et al., 2020). If the livestock is stressed or overactive before slaughter, beef glycogen content and secreted lactic acid will be reduced, resulting in an increase in the final pH of the muscle to between 5.8 and 6.0, meaning the beef will be tough (Picard & Gagaoua, 2020).

Rump beef is in low demand because of its low‐fat content and dry texture. However, value can be added by enhancing taste and flavor by aging non‐preferred parts. Ripening improves the flavor, mouth feel, and juiciness of beef (Irurueta et al., 2008). Ripening can involve either dry aging or wet aging, depending on the chosen method. Dry aging involves a maturation process in which beef is kept in the fridge; normally, the humidity is approximately 88%, with temperatures maintained between −1 and 2℃. During wet aging, the beef is kept in a vacuum‐packed state (Smith et al., 2008; Tikk et al., 2006) to prevent oxidation and moisture evaporation during storage; this prevents the growth of aerobic bacteria (Smith et al., 2008).

During aging, enzymes from the beef itself decompose proteins, carbohydrates, and hexane (Meinert et al., 2009). This decomposition reaction results in the generation of low‐molecular water‐soluble odor precursors such as amino acids, peptides, non‐protein nitrogen compounds, monosaccharides, and phosphate compounds, thereby enhancing the odor of beef (Koutsidis et al., 2003; Mottram, 1998). Amino acids generate volatile odor components through Maillard and Strecker reactions (Majcher & Jeleń, 2007). In particular, free amino acids directly participate in beef taste and indirectly produce various odors (Mullen et al., 2000). Odor components are also produced by microbial fermentation (Daszkiewicz et al., 2003; Miller et al., 1997; Picard & Gagaoua, 2020).

The Korean Food Code stipulates that volatile basic nitrogen (VBN) levels should be ≤20 mg/100 g for both raw and packaged beef. Although monitoring the bacterial diversity and the chemical composition of the meat and volatile organic compounds (VOCs) during storage has been previously discussed (Smith et al., 2008), the correlation between VBN and other parameters in cold‐stored aged beef is yet to be reported in the literature.

This study aimed to analyze the composition of the microorganism community, volatile compounds, and free amino acids in aged beef during cold storage and to correlate profiles of physicochemical properties in aged beef with VBN levels, which might lead to an enhancement of the quality of the aged beef model. As a result of this study, it was possible to provide guidelines regarding the optimum method of storage for each type of aged meat depending on the basic data of the change in the quality of aged meat during refrigerated storage.

## 2 MATERIALS AND METHODS

### 2.1 Materials

Wet‐aged, dry‐aged, and raw beef (rump) were purchased from a local grocery market (Jeollabuk‐do, Korea). PrepMan Ultra Sample preparation reagent was purchased from Applied Biosystems. Sulfuric acid (0.02 N), sodium hydroxide (0.01 N), and potassium sulfate were purchased from Samchun Chemical Co. Volatile compound test internal standard (3,3‐dimethyl‐2‐butanol) and trichloroacetic acid were purchased from Sigma‐Aldrich. The amino acid mixture standard solutions, including Type B and Type AN‐Ⅱ, were purchased from Wako Pure Chemical Industries Ltd.

### 2.2 Sampling and storage of beef

Beef rump samples were placed in an icebox and transported to the laboratory within 2 h of purchase. In the laboratory, samples were divided into 0.2 kg portions, put in a permeable polyethylene bag (200 × 300 mm), and stored in a refrigerator at 4°C (LG CA‐H17DZ) for 7‐, 14‐, and 21‐day sampling. The total sample was prepared in three packages, each weighing 0.2 kg. On each sampling day, beef specimens were ground according to the experimental method.

### 2.3 Microbiological analysis

#### 2.3.1 Quantitative microbiological analysis

The beef samples were serially diluted with sterilized 0.85% saline solution to determine the viable bacterial counts. Bacterial counts were measured using 3 M Petrifilm count plates (3 M‐UK), according to the manufacturer's instructions as follows: 3 M Petrifilm AC plates for aerobic mesophilic bacterial counts; 3 M Petrifilm LAB plates for lactic acid bacteria count LAB; 3 M Petrifilm *Escherichia coli*/Coliform Count Plates for coliform counts; and 3 M Petrifilm Yeast and Mold Count Plates for yeast. Psychrophilic bacteria, mesophilic bacteria, coliform bacteria, and *Escherichia coli* were incubated on principal component analysis (PCA) (Merck) plates at 35°C for 2–3 days. Psychrophilic bacteria, LAB, yeast, and mold were incubated at 25°C for 2–3 days, respectively. Colonies were counted from 3 M films, on which 30–300 colonies appeared, and were reported as log CFU/ml.

#### 2.3.2 Identification of microorganisms using matrix‐assisted laser desorption ionization–time‐of‐flight mass spectrometry (MALDI‐TOF MS)

Identification of microorganisms using VITEK MS (Biomeriux) was performed using the direct colony method according to the manufacturer's suggestion. A raw colony obtained from three additional subcultures was smeared onto a spot of a MALDI disposable target plate, which was then covered with 1 μl of VITEK MS‐CHCA (Biomeriux). After drying, the target plate was loaded into the VITEK MS platform according to the default settings. *E. coli* ATCC 8739 was used for instrument calibration and quality control.

#### 2.3.3 Identification of microorganisms using 16S rRNA sequencing

All colonies were selected from the highest dilution PCA spread plates, which usually contained 30–100 isolates using an inoculation loop and sub‐culture at 30°C for 24–48 h. All colonies were isolated and purified using PrepMan ultra sample preparation reagents. Genomic DNA was diluted 1:500 in nuclease‐free water. Polymerase chain reaction (PCR) and cycle sequencing were performed according to the manufacturer's manual using the Fast MicroSeq 500 Bacterial Identification kit (Applied Biosystems) with a PCR machine (Biometra). Extension and sequencing clean‐up were performed with the ExoSAP‐IT (USB Products, Affymetrix, Inc.) and Performance DTR cartridges (Edge BioSystems), respectively, according to the manufacturer's instructions. Sequences were detected on a 3500xL Genetic Analyzer (Applied Biosystems) with POP‐7 polymer (Applied Biosystems). Analysis of sequencing data was performed using the MicroSEQ ID Software (Ver. 2.2).

### 2.4 Chemical analysis

#### 2.4.1 Measurement of pH and VBN

Ten grams of sample and 40 ml of distilled water were added to a mixer and homogenized. The sample’s pH was measured using an 827 pH lab meter (Metrohm AG). The homogenized sample was filtered, and the solution was decanted into a 100‐ml volumetric flask. One milliliter of 0.02 N H_2_SO_4_ and 1–2 drops of the VBN indicator were added to the inner chamber of the Conway unit, while 1 ml of the sample and 1 ml of saturated K_2_CO_3_ were added to the outside chamber. Glycerin was slightly applied to the border of the cover, which was immediately sealed; the sample was gently stirred in the horizontal direction and left at 25°C for at least 60 min. Thereafter standing, the inner chamber was titrated with 0.01 M NaOH, until the liquid turned light green.

#### 2.4.2 VOC analysis

Two grams of sample was placed into 20‐ml headspace vials; 25 μl of the internal standard solution (100 μg/ml 3,3‐dimethyl‐2‐butanol) was slowly added into each vial, and the lid was closed with a polytetrafluoroethylene/silicone septum (Supelco; Sigma‐Aldrich). Three replicates were analyzed for each sample. The sample was equilibrated at 50°C for 30 min and extracted using carboxen/polydimethylsiloxane (Supelco; Sigma‐Aldrich) in the headspace for 30 min. The fiber was desorbed in a gas chromatograph injector at a temperature of 220°C for 30 s.

The analytical equipment used was a GC‐TOF/MS (GC: Agilent 6890N, Agilent Technologies, TOF/MS: LECO Corp.). The primary column was DB‐WAX (60 m × 0.25 mm internal diameter (ID) × 0.25 µm film thickness), and the secondary column was DB‐1701 (20 m × 0.10 mm ID × 0.40 μm film thickness). The primary column temperature was programmed from 40°C for 2 min to 230°C at a rate of 20°C/min and held for 10 min. The second column was always operated at 10°C higher than the primary column temperature. The GC carrier gas used high‐purity helium and a gas flow rate of 1.2 ml/min. The inlet temperature was 220°C and the spilt ratio was 20:1. The detector voltage was 1600 V, and the electron energy was 70 eV. The acquisition delay time was 180 s, and the data were collected in SCAN mode (33–650 m/z). The ion source temperature was 200°C. The software ChromaTOF^®^ (VECO Corp, version 4.22) was used for data analysis. Only peaks with a signal‐to‐noise ratio of 20 were extracted. For the identification of compounds, the compound's mass spectrum was compared with the library mass spectra database (NIST and Wiley), with a minimum match score of 800. The unique mass of each detected volatile compound was selected, and the area of each peak was calculated.

#### 2.4.3 Free amino acid analysis

Standard amino acid mixtures, including Type B and Type AN‐II, were used to quantify the free amino acid contents. Five grams of sample was weighed and homogenized with 15 ml of 5% trichloroacetic acid, followed by centrifugation at 10,000 *g* at 4 ℃ for 15 min in a HITACHI CR21GⅢ centrifuge. Five milliliters of the supernatant and 5 ml of hexane were mixed and vortexed. Then, 1 ml of the lower layer was taken and filtered using a 0.2‐μm filter. Three replicates were analyzed for each sample.

The analytical equipment used was the Amino Acid Automatic Analyzer L‐8900 (Hitachi). The guard column was AN0‐9256, the main column was a PF column (488511), and the ammonia filter column was 19664. The column temperature ranged from 30 to 70°C (increase 1°C/step). The reactor heater temperature was 135°C. The visible detector wavelengths were 570 nm and 440 nm (for proline). The column flow rate was 0.35 ml/min. The total analysis time was 157.3 min.

The amount of each amino acid in the samples was calculated with reference to the standard sample using the EZChrom Elite (Hitachi High‐Technologies Corporation, 2004) software, and the content of each amino acid was expressed as a percentage of the total sample weight. Determinations were made with three replications for each sample.

### 2.5 Statistical analysis

Data obtained from quantitative microbiological analyses were transformed to log values (log CFU/ml) prior to statistical analysis. The average, standard deviation, and minimum and maximum values of the data were calculated for each factor and group. The correlation coefficient between the VOCs data and free amino acid data was calculated using Spearman's correlation and PCA. Statistical analysis was performed using the IBM SPSS software (v.20; SPSS Inc.) and SIMCA software (v.16; MKS Data Analytics Solutions). Statistical significance was identified at *p* < .05 and *p* < .001.

## 3 RESULTS AND DISCUSSION

### 3.1 Microbial growth

**FIGURE 1:**
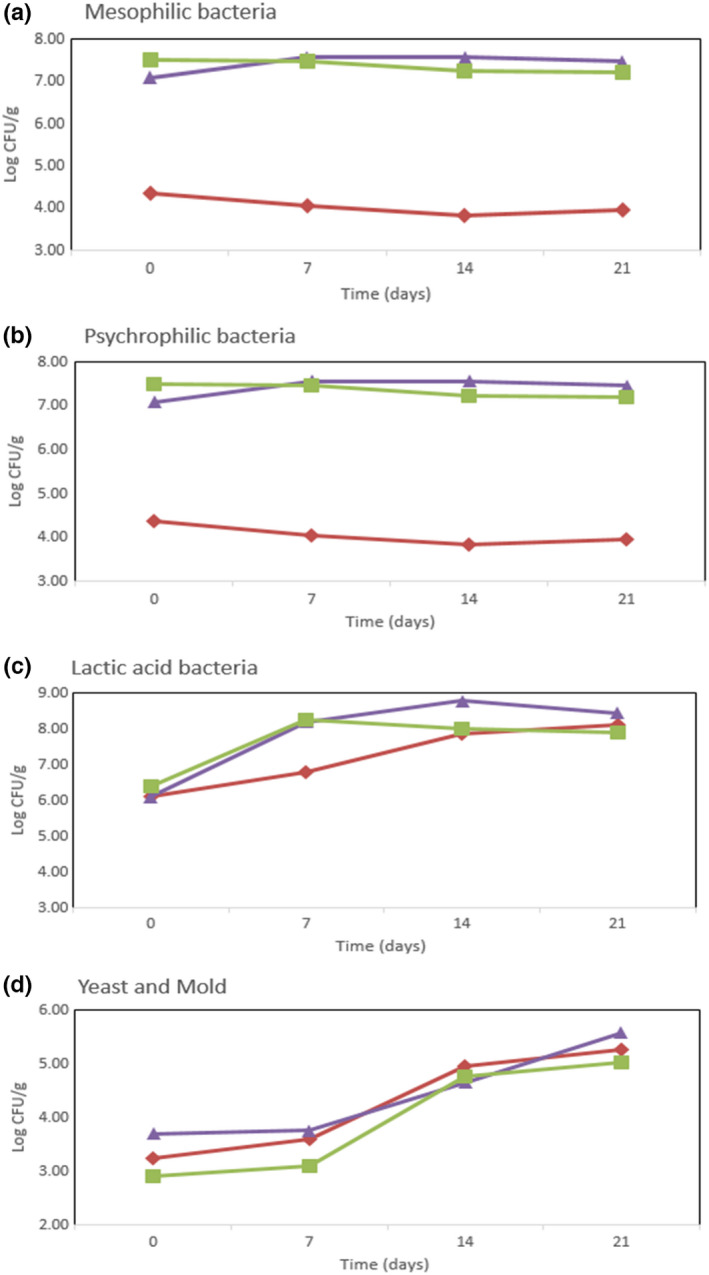
Enumeration (CFU/g) of (a) mesophilic, (b) psychrophilic, and (c) lactic acid bacteria; and yeast/mold on different cold‐stored beef preparations (21 days)

The viable counts of different microbial groups in raw beef (a), wet‐aged beef (b), and dry‐aged beef (c) during 21 days of cold storage are shown in Figure 1. The raw beef samples stored for 21 days showed viable counts ranging 4.37–3.95 log CFU/g of mesophilic bacteria, 5.75–7.78 log CFU/g of psychrophilic bacteria, 6.10–8.12 log CFU/g of lactic acid bacteria, and 3.24–5.26 log CFU/g of yeast and mold. The length of the storage period had no effect on the number of mesophilic bacteria. However, the initial values of psychrophilic bacteria and lactic acid bacteria were higher than 5 log CFU/g and were more than 2 log CFU/g after 21 days. The wet‐aged beef samples stored for 21 days showed viable counts ranging 7.50–7.20 log CFU/g of mesophilic bacteria, 8.33–7.80 log CFU/g of psychrophilic bacteria, 6.42–7.90 log CFU/g of lactic acid bacteria, and 2.91–5.04 log CFU/g for yeast and mold. With the exception of yeast and mold, there was either no change or a decrease in the number of bacteria as the storage time increased. The dry‐aged beef samples stored for 21 days showed values similar to wet‐aged beef, with viable counts ranging 7.09–7.47 log CFU/g of mesophilic bacteria, 8.28–7.98 log CFU/g of psychrophilic bacteria, 6.10–8.46 log CFU/g of lactic acid bacteria, and 3.70–5.59 log CFU/g for yeast and mold.

Microbial growth in beef depends on the environmental conditions during the aging process, ultimately impacting beef spoilage and quality deterioration. Therefore, controlling microbial growth during storage is vital (Campbell et al., 2001; Kim et al., 2019). The spoilage of raw beef is mainly caused by undesired microbial development in beef during storage. The type of bacteria and their loads depend on the initial beef contamination and on the specific storage conditions. The storage conditions can influence the development of different spoilage‐related microbial populations, thus affecting the type and rate of spoilage (Ellis et al., 2004).

### 3.2 Microbial analysis

**FIGURE 2:**
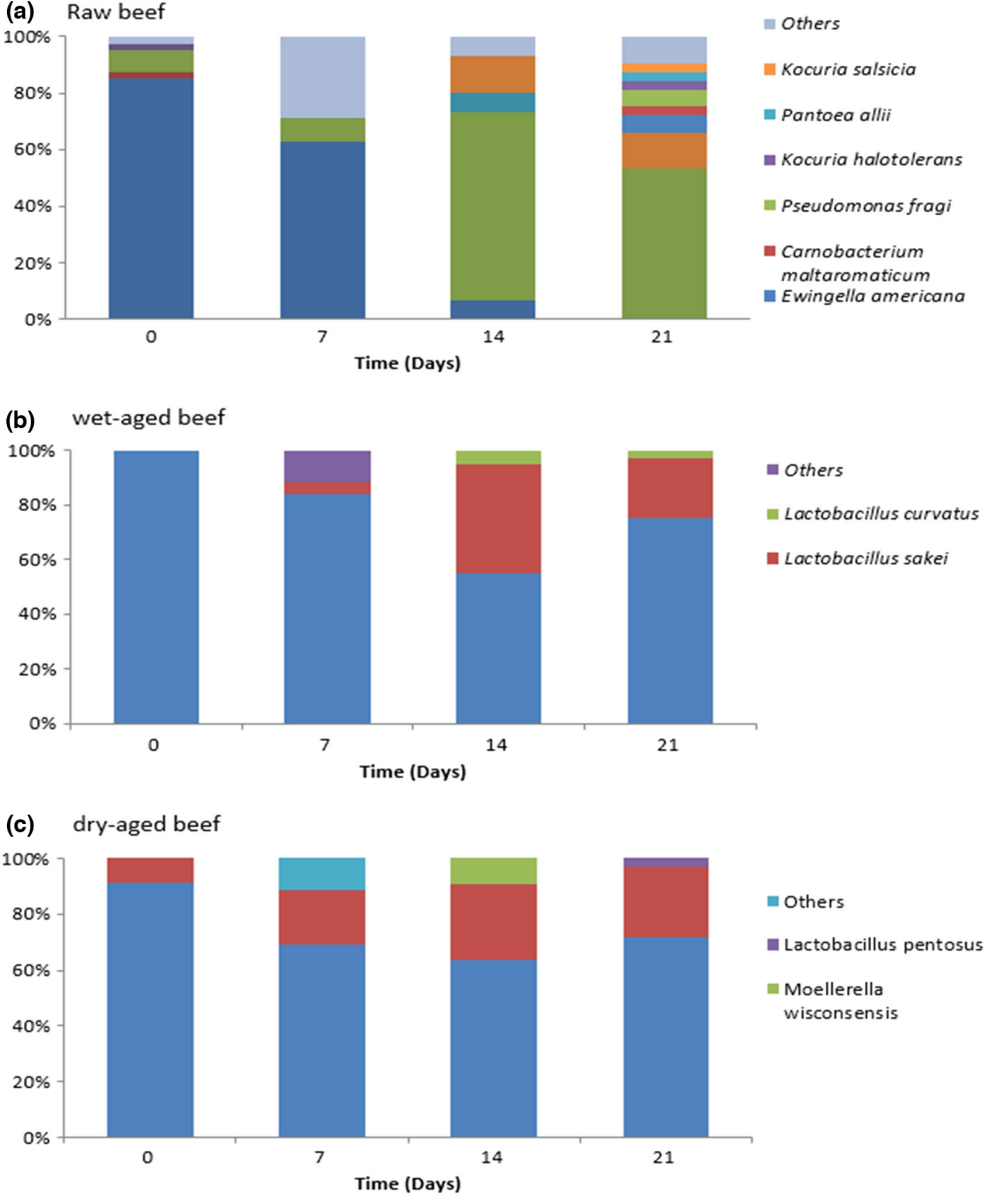
Utilizing amplicon next‐generation sequencing of 16S rRNA in determining the genetic composition of microbiota in different cold‐stored beef preparations

Randomly selected colonies isolated on PCA plates at different storage days were identified, and their relative abundance (%) of each bacterial species are shown in Figure 2. Raw beef was initially dominated by *Staphylococcus saprophyticus,* and the levels of *S. saprophyticus* decreased with longer cold storage times. *Pseudomonas fragi* became the dominant bacteria in raw beef after 21 days. *P. fragi* is recognized as the dominant spoiler species in beef and is the main species responsible for spoilage under aerobic conditions (Argyri et al., 2015; Casaburi et al., 2015). *P. fragi* showed a positive correlation with octanal, nonanal, and alcohols, while *Carnobacterium divergens* and *Lactococcus piscium* were correlated with ethyl lactate, ethyl acetate, and medium‐chain fatty acids (Botta et al., 2018).

The dominant bacteria in both wet‐ and dry‐aged beef was *C. divergens*, and as time passed, the levels of *Lactobacillus sakei* gradually increased. Both lactic acid bacteria and *Brochothrix thermosphacta* are causes of spoilage; their presence can be characterized by souring rather than putrefaction (Kuuliala et al., 2018; Nychas et al., 2008). The different microbial groups that will potentially contribute to beef spoilage depend on the storage conditions applied and their competition. In general, the metabolic activity of the ephemeral microbial association, which prevails in a beef ecosystem under certain aerobic conditions or generally introduced during processing, leads to the manifestation of changes or spoilage of beef (Nychas et al., 2008). The environment then enforces a selective pressure on the bacterial community, and those groups of bacteria best adapted to the environment will outgrow the others, become dominant, and reach high numbers. Thus, the survival, growth, and succession of specific spoilage bacteria can be affected by the diversity of ecophysiological factors in the physical and chemical environment. These factors, including beef constituents, temperature, pH, oxygen or carbon dioxide (packaging atmosphere), and competing microbiota, are important for maintaining beef quality over time (Doulgeraki et al., 2012). Although the role of bacteria in the development of spoilage‐associated molecules during meat storage is being recognized, data are still lacking on the VOCs profile of stored meat and its possible association with microbial species acting as spoilage factors (Ercolini et al., 2009).

### 3.3 Physical parameters analysis

The physical parameters, including pH and VBN changes, in raw, wet‐aged, and dry‐aged beef preparations at 4°C are shown in Table 1. The initial pH in the raw, wet‐aged, and dry‐aged beef preparations were 5.52, 5.60, and 5.59, respectively. During cold storage, the pH of raw, wet‐aged, and dry‐aged beef preparations gradually decreased to 5.04, 5.23, and 5.33, respectively. The pH of raw beef decreased the most compared to the other samples during cold storage, which is consistent with previous reports (Holley et al., 1994). The accumulation of peroxides from organic acids, aldehydes, ketones, alcohol, and carbonyls due to fat burning and the breakdown of sugars and fats may have a role in lowering pH levels in red beef (Tak et al., 2005).

**TABLE 1:** Changes in pH and volatile basic nitrogen (VBN) in aged beef during cold storage

Samples	Storage time (days)	pH	VBN^1^ (mg/100 g)
Raw beef	0	5.52	7.56 ± 0.28^2^ d
	7	5.44	9.80 ± 0.16 c
	14	5.25	13.72 ± 2.51 b
	21	5.04	22.59 ± 0.43 a
Wet‐aged beef	0	5.60	2.71 ± 0.43 d
	7	5.54	5.04 ± 0.30 c
	14	5.44	8.68 ± 0.07 b
	21	5.23	20.53 ± 0.32 a
Dry‐aged beef	0	5.59	6.07 ± 0.28 d
	7	5.45	7.84 ± 0.16 c
	14	5.50	10.55 ± 2.59 b
	21	5.33	20.91 ± 0.43 a

Different letters (a,b,c,d) indicate significant differences at 0.05.

^1^ The Korean Food Code stipulates that VBN is 20 mg/100 g or less for both raw and packaged beef.

^2^ Data are presented as mean ± standard deviation (*n* = 3).

The initial VBN value of raw beef was 7.56 mg/100 g, which increased to 22.59 mg/100 g with increasing storage period. The wet‐aged beef increased from 2.71 to 20.53 mg/100 g, while that of the dry‐aged beef ranged from 6.07 to 20.91 mg/100 g. Especially, wet‐aged beef's initial VBN value reached the decay standard the fastest (velocity = 0.85). The Korean Food Code stipulates that VBN levels should be ≤20 mg/100 g for both raw and packaged beef. Therefore, raw, wet‐aged, and dry‐aged beef preparations all exceed the standard after 21 days and can be considered to have started the spoilage process.

### 3.4 Changes in volatile compounds and free amino acids

The changes in the volatile components of raw, wet‐aged, and dry‐aged beef preparations maintained at 4°C are shown in Table 2. The volatiles were semi‐quantified by dividing the peak area of the compound of interest by the peak area of the internal standard (100 μg/ml 3,3‐dimethyl‐2‐butanol). Fourteen volatiles, including dimethyl sulfide, four ketones, six alcohols, 3‐methylbutanal, and two acids, were detected in the beef samples. Before storage, the volatiles with the highest levels in raw beef, wet‐aged beef, and dry‐aged beef were 3‐hydroxy‐2‐butanone and ethanol, respectively. With the exception of dimethyl sulfide, the level of other volatiles in the three beef preparations increased over time. A noticeable increase in ketones and alcohols was observed in raw beef; 2,3‐butanedione levels increased by more than 1200 times, while those of 1‐hexanol increased by more than 141 times. The only significant changes in dry‐aged beef are ethanol and 3‐methylbutanal levels, which increased by more than 2 and 18 times, respectively, after 21 days of cold storage. In wet‐aged beef, the levels of most volatile compounds were lower than those in raw and dry‐aged beef. However, 2,3‐butanedione and 1‐butanol levels were seven‐ and five‐fold higher, respectively, than those of dry‐aged beef after 21 days of cold storage.

**TABLE 2:** Changes in volatile organic compounds (VOCs) in aged beef during cold storage

Functional group/Compound	Retention Time (min)	Raw beef (days)			Wet‐aged beef (days)			Dry‐aged beef (days)		
		0	7	21	0	7	21	0	7	21
Sulfur compounds										
Dimethyl sulfide	7.24	0.06 ± 0.02^1^ ^,^ ^2^	0.04 ± 0.01	0.03 ± 0.00	0.03 ± 0.01	0.02 ± 0.00	0.01 ± 0.00	0.02 ± 0.02	0.01 ± 0.00	0.01 ± 0.00
Ketones										
2‐Butanone	8.30	0.01 ± 0.00	0.02 ± 0.00	0.05 ± 0.00	0.03 ± 0.00	0.09 ± 0.02	0.32 ± 0.02	0.04 ± 0.02	0.06 ± 0.00	0.38 ± 0.08
2,3‐Butanedione	9.07	0.12 ± 0.02	7.39 ± 3.13	147.23 ± 3.14	1.30 ± 0.52	16.87 ± 0.32	22.97 ± 1.06	0.47 ± 0.14	4.17 ± 0.73	3.46 ± 1.08
2‐Heptanone	10.59	0.03 ± 0.01	0.02 ± 0.01	0.31 ± 0.02	0.00 ± 0.00	0.01 ± 0.00	0.03 ± 0.01	0.01 ± 0.01	0.01 ± 0.01	0.03 ± 0.01
3‐hydroxy‐2‐butanone	11.49	1.72 ± 0.41	13.69 ± 4.69	58.60 ± 43.11	3.13 ± 0.97	20.31 ± 1.27	22.80 ± 1.90	2.21 ± 1.68	21.61 ± 5.38	53.82 ± 19.61
Alcohols										
Ethanol	8.44	0.01 ± 0.01	0.02 ± 0.00	0.22 ± 0.01	0.22 ± 0.02	0.37 ± 0.01	0.27 ± 0.09	7.73 ± 6.79	11.98 ± 0.23	16.45 ± 1.71
1‐Butanol	10.33	0.04 ± 0.01	0.03 ± 0.00	0.17 ± 0.01	0.07 ± 0.01	0.11 ± 0.01	0.09 ± 0.01	0.03 ± 0.01	0.02 ± 0.00	0.02 ± 0.00
1‐Pentanol	11.21	0.92 ± 0.15	0.60 ± 0.10	4.85 ± 0.39	0.39 ± 0.07	0.38 ± 0.02	0.41 ± 0.02	0.32 ± 0.40	0.23 ± 0.02	0.12 ± 0.07
1‐Hexanol	12.04	0.15 ± 0.02	0.31 ± 0.13	21.29 ± 1.73	0.15 ± 0.13	0.62 ± 0.03	0.99 ± 0.15	0.06 ± 0.05	0.24 ± 0.03	0.80 ± 0.88
1‐Octen‐3‐ol	12.41	0.22 ± 0.04	0.17 ± 0.07	1.36 ± 0.17	0.13 ± 0.03	0.20 ± 0.03	0.39 ± 0.01	0.08 ± 0.08	0.08 ± 0.01	0.13 ± 0.15
2,3‐Butanediol	13.31	0.01 ± 0.00	0.01 ± 0.00	0.38 ± 0.17	0.05 ± 0.01	0.01 ± 0.00	0.19 ± 0.09	0.07 ± 0.05	0.03 ± 0.00	1.59 ± 0.42
Aldehyde										
3‐Methylbutanal	8.42	nd	nd	0.33 ± 0.25	0.01 ± 0.00	0.02 ± 0.00	0.14 ± 0.06	0.60 ± 0.52	2.16 ± 0.26	10.97 ± 2.92
Acids										
Acetic acid	12.43	0.09 ± 0.03	0.45 ± 0.20	1.95 ± 0.41	0.08 ± 0.03	0.62 ± 0.09	0.43 ± 0.23	0.15 ± 0.06	1.13 ± 0.23	0.59 ± 0.29
Butanoic acid	13.50	0.03 ± 0.01	0.07 ± 0.03	0.14 ± 0.03	0.01 ± 0.00	0.05 ± 0.00	0.03 ± 0.01	0.04 ± 0.01	0.16 ± 0.04	nd

Abbreviation: nd, not detected.

^1^ The volatile compounds were semiquantified by dividing the peak areas of the compounds of interest by the peak area of internal standard (100 μg/ml 3,3‐dimethyl‐2‐butanol)

^2^ Data are presented as mean ± standard deviation (*n* = 3).

Free amino acid changes in raw, wet‐aged, and dry‐aged beef preparations at 4°C are shown in Table 3. The initial contents of all free amino acids in dry‐aged beef were higher than those in raw and wet‐aged beef. Before storage, the highest content of a free amino acid in raw, wet‐aged, and dry‐aged beef was that of l‐alanine.

**TABLE 3:** Changes in free amino acids in aged beef during cold storage

Free amino acids	Raw beef (days) (µg/ml)			Wet‐aged beef (days) (µg/ml)			Dry‐aged beef (days) (µg/ml)		
	0	7	21	0	7	21	0	7	21
Aspartic acid	0.46 ± 0.01^1^	0.16 ± 0.00	0.42 ± 0.02	0.24 ± 0.01	0.43 ± 0.03	0.68 ± 0.15	4.23 ± 0.04	4.67 ± 0.07	7.54 ± 1.29
Threonine	1.72 ± 0.02	1.96 ± 0.05	3.04 ± 0.10	2.70 ± 0.13	3.51 ± 0.26	5.04 ± 1.08	13.54 ± 0.25	15.62 ± 0.16	12.67 ± 2.16
Serine	2.15 ± 0.02	2.62 ± 0.06	3.25 ± 0.11	4.02 ± 0.08	4.54 ± 0.35	6.24 ± 1.33	16.90 ± 0.32	18.76 ± 0.18	9.55 ± 1.67
Glutamic acid	3.94 ± 0.06	6.23 ± 0.19	5.13 ± 0.22	5.49 ± 0.18	6.99 ± 0.72	7.09 ± 1.30	26.93 ± 0.50	30.69 ± 0.36	23.21 ± 3.98
Glycine	3.27 ± 0.03	3.46 ± 0.08	4.12 ± 0.15	4.58 ± 0.08	4.60 ± 0.42	5.84 ± 1.27	11.68 ± 0.17	13.54 ± 0.13	9.23 ± 1.60
Alanine	11.73 ± 0.13	12.51 ± 0.32	17.00 ± 0.58	11.84 ± 0.29	13.40 ± 1.09	19.3 ± 4.10	32.33 ± 0.47	40.59 ± 0.55	29.07 ± 5.15
Valine	1.77 ± 0.02	2.55 ± 0.06	5.28 ± 0.15	3.64 ± 0.06	4.97 ± 0.49	7.89 ± 1.72	18.34 ± 0.28	21.35 ± 0.21	18.65 ± 3.23
Cysteine	nd	nd	nd	0.00 ± 0.01	0.01 ± 0.00	0.07 ± 0.11	0.09 ± 0.03	0.30 ± 0.01	0.73 ± 0.12
Methionine	0.93 ± 0.01	1.67 ± 0.05	3.14 ± 0.10	2.17 ± 0.06	3.07 ± 0.23	4.30 ± 0.83	9.50 ± 0.22	10.51 ± 0.08	8.22 ± 1.44
Isoleucine	1.12 ± 0.02	1.52 ± 0.04	3.08 ± 0.11	2.49 ± 0.15	3.48 ± 0.26	5.49 ± 1.17	14.17 ± 0.80	14.92 ± 0.14	11.8 ± 2.07
Leucine	2.16 ± 0.03	3.85 ± 0.10	8.45 ± 0.27	4.76 ± 0.11	7.69 ± 0.59	10.83 ± 2.32	24.52 ± 1.01	32.34 ± 0.30	19.38 ± 3.43
Tyrosine	1.21 ± 0.02	1.13 ± 0.04	0.06 ± 0.00	0.95 ± 0.02	0.34 ± 0.03	0.19 ± 0.04	4.91 ± 034	2.68 ± 0.02	1.01 ± 0.20
Phenylalanine	1.49 ± 0.02	2.07 ± 0.06	3.66 ± 0.13	3.03 ± 0.09	4.00 ± 0.29	5.02 ± 1.03	14.30 ± 0.29	15.90 ± 0.19	9.58 ± 1.66
Lysine	1.96 ± 0.03	2.48 ± 0.06	3.63 ± 0.13	3.57 ± 0.08	3.05 ± 0.24	3.20 ± 0.70	22.10 ± 0.34	25.41 ± 0.16	19.27 ± 3.25
Histidine	nd	0.06 ± 0.00	0.05 ± 0.01	0.04 ± 0.00	0.08 ± 0.01	0.09 ± 0.02	0.02 ± 0.00	nd	0.06 ± 0.11
Arginine	2.19 ± 0.02	2.33 ± 0.06	0.01 ± 0.00	2.63 ± 0.12	0.34 ± 0.03	0.04 ± 0.01	15.55 ± 0.44	11.33 ± 0.09	0.36 ± 0.07
Proline	0.90 ± 0.02	0.96 ± 0.07	1.10 ± 0.11	1.66 ± 0.03	1.61 ± 0.16	2.42 ± 0.57	7.03 ± 0.10	7.14 ± 0.15	4.45 ± 0.78

Abbreviation: nd, not detected.

^1^ Data are presented as mean ± standard deviation (*n* = 3).

Generally, levels of free amino acids in raw and wet‐aged beef increased with storage time, while those in dry‐aged beef decreased with storage time. l‐arginine and l‐tyrosine decreased in all beef preparations. In dry‐aged beef, l‐arginine decreased more than 43‐fold compared to raw and wet‐aged beef. The levels of most of the free amino acids tended to decrease in dry‐aged beef, with the exception of l‐aspartic acid, l‐cysteine, and l‐histidine.

The contents of amino acids gradually increased as the Z‐disks slowly collapsed during aging (Monsón et al., 2004). However, as in the case of dry‐aged beef in this experiment, when the free amino acid content is very high, it shows a tendency to decrease with time. The degradation enzymes involved in aging might be involved in decreasing free fatty acid levels in dry‐aged beef.

**FIGURE 3:**
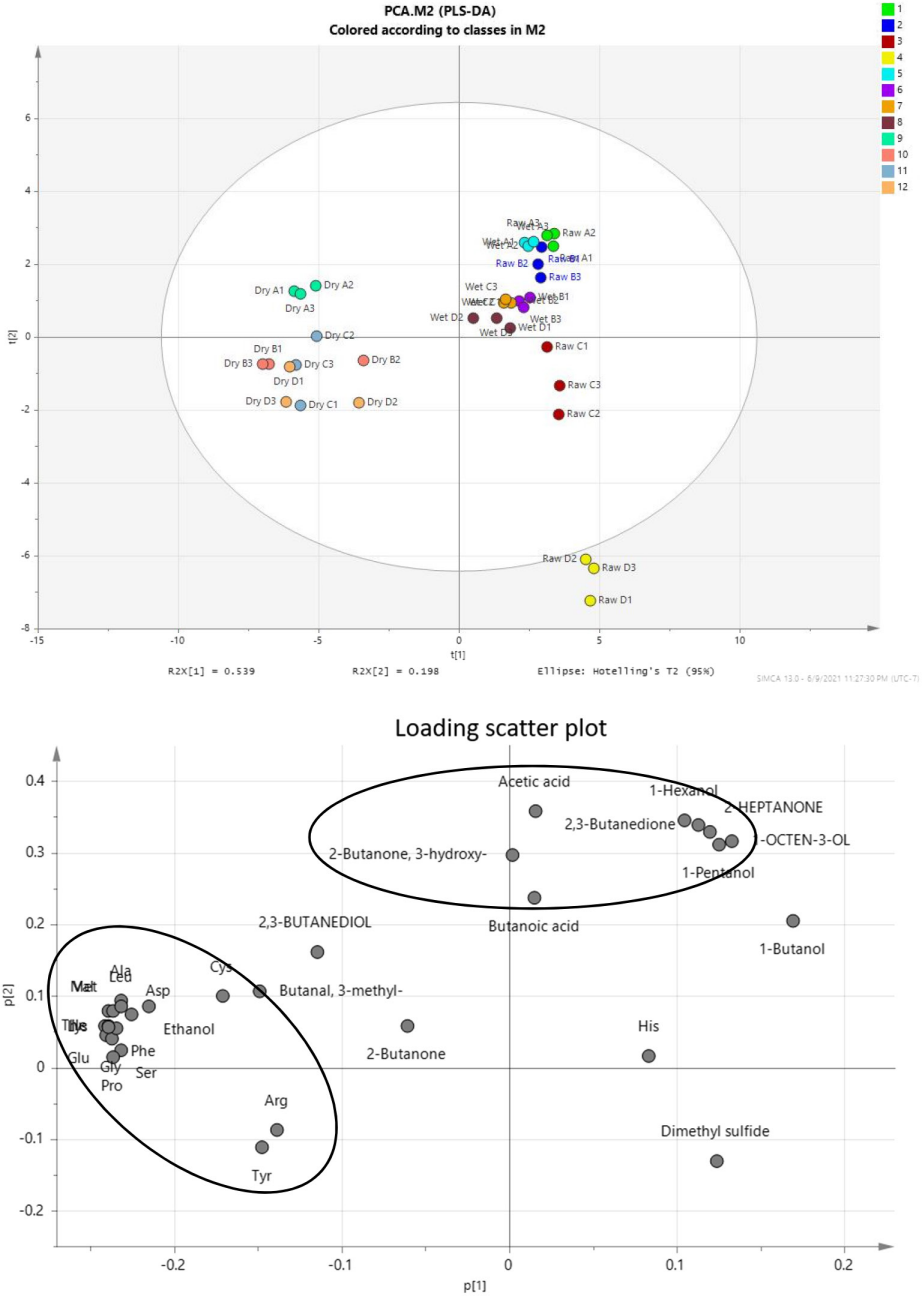
Principal component analysis of raw and aged beef during cold storage using profiles of VOCs and free amino acids

Principal component analysis of raw and aged beef during cold storage using profiles of VOCs and free amino acids are shown in Figure 3. PCA demonstrated a correlation between raw beef and 2,3‐butanedione and 3‐hydroxy‐2‐butanone. The compounds 2,3‐butanedione and 1‐hexanol showed a strong correlation with wet‐aged beef, while l‐aspartic acid and l‐cysteine showed a strong correlation with dry‐aged beef.

According to the correlation analysis among the volatile compounds and free amino in raw beef, the amino acids, l‐serine, glycine, l‐alanine, l‐valine, l‐methionine, l‐isoleucine, l‐leucine, l‐phenylalanine, and l‐lysine, and volatile compounds, butanone, ethanol, hexanol, acetic acid butanediol, and butanoic acid, were found to have increased over time. Furthermore, l‐tyrosine tended to decrease with butanone, ethanol, hexanol, acetic acid, butanediol, and butanoic acid, while l‐arginine tended to decrease with butanol, heptanone, pentanol, and octenol.

l‐aspartic acid and l‐isoleucine tended to increase with the levels of butanone, butanedione, butanal methyl, hexanol, and octenol over time in wet‐aged beef. l‐alanine, l‐valine, and methyl butanal have a correlation coefficient of 0.9. In contrast, l‐tyrosine and l‐arginine levels tended to decrease with time, along with butanone, butanedione, methyl butanal, hexanol, and octenol.

In dry‐aged beef, l‐aspartic acid tended to increase with butanone, butanedione, and butanal methyl over time, while l‐serine tended to decrease with butanone. Butanediol and l‐arginine tended to decrease with butanone, butanal methyl, and hexenol.

Free amino acids subsequently generate volatile compounds through Maillard and Strecker Reactions (Meinert et al., 2007, 2009). Aldehydes in volatile compounds are produced through the automatic oxidation of fatty acids. These compounds have a strong effect on the odor of beef products, even at low concentrations, and they produce odors such as butter, sweet, floral, and green (Ozkara et al., 2019). 3‐Methylbutanal and 2‐methylpropanal are produced through the decomposition of l‐isoleucine and l‐valine, while benzeneacetaldehyde is produced through the Strecker degradation of l‐phenylalanine (Škrlep et al., 2019). Straight‐chain ketones are also produced by fatty oxidation (Yasuhara & Shibamoto, 1989), and 1‐hydroxy‐2‐propanone, 3‐hydroxy‐2‐butanone, acetoin (3‐hydroxy‐2‐butanone), and 3‐methyl‐2‐pentanone are produced by microbial fermentation of glucide (Xiao et al., 2012). 2‐Butanone and other methyl‐ketones are produced by β‐oxidation of saturated fatty acids by microorganisms (Sunesen et al., 2001). Nitrogen and sulfide compounds in the volatile compounds are mainly derived from amino acids. In particular, sulfide compounds are caused by the reaction between hydrogenated amino acids such as l‐cysteine, l‐methionine, and carboxylic compounds produced from fatty acids (Goeke, 2002). Dominant microorganisms might have a critical role in the production of free amino acids and headspace volatiles in cold‐stored beef, which could affect VBN levels. This study investigates microorganisms, free fatty acids, and headspace volatiles as some of the major factors leading to the deterioration of the quality of cold‐stored beef.

## 4 CONCLUSION

Profile changes in microorganisms, volatile compounds, and free amino acids in cold‐stored raw, wet‐aged, and dry‐aged beef preparations had correlations with each other. During cold storage, both psychrophilic and lactic acid bacteria increased in raw beef, while mesophilic, psychrophilic, and lactic acid bacteria decreased in dry‐aged and wet‐aged beef. Alterations in the composition of microorganism communities led to changes in the profiles of volatiles and amino acids in raw, dry‐aged, and wet‐aged cold‐stored beef preparations. 2,3‐Butanedione, 2‐butanone, tyrosine, and arginine seemed to have a role in reaching spoilage levels in cold‐stored beef irrespective of the treatment type (raw, dry aging, and wet aging). The current study provides basic information on the process of evaluating aging beef quality, potentially benefiting the beef industry as a whole and boosting consumer acceptance.

## CONFLICT OF INTEREST

No potential conflicts of interest were reported by the authors.
